# Predictors of survival and toxicity in patients on adjuvant therapy with 5-fluorouracil for colorectal cancer

**DOI:** 10.1038/sj.bjc.6605052

**Published:** 2009-04-21

**Authors:** M Gusella, A C Frigo, C Bolzonella, R Marinelli, C Barile, A Bononi, G Crepaldi, D Menon, L Stievano, S Toso, F Pasini, E Ferrazzi, R Padrini

**Affiliations:** 1 Laboratory of Pharmacology and Molecular Biology, Oncology Department, Azienda-ULSS 18-Rovigo, Via Ugo Grisetti 265, 45027 Trecenta, Italy; 2 Department of Environmental Medicine and Public Health, University of Padova, via Loredan 18, 35131 Padova, Italy; 3 Medical Oncology, Oncology Department, Azienda ULSS 18-Rovigo, Viale Tre Martiri 89, 45100 Rovigo, Italy; 4 Department of Clinical and Experimental Medicine, University of Padova, via Giustiniani 2, 35128 Padova, Italy

**Keywords:** fluorouracil, adjuvant therapy, colorectal cancer, pharmacokinetics, pharmacogenetics.

## Abstract

The present study aimed at investigating whether the simultaneous evaluation of pharmacokinetic, pharmacogenetic and demographic factors could improve prediction on toxicity and survival in colorectal cancer patients treated with adjuvant 5-fluorouracil (5FU)/leucovorin therapy. One hundred and thirty consecutive, B2 and C Duke's stage colorectal cancer patients were prospectively enrolled. 5FU pharmacokinetics was evaluated at the first cycle. Thymidylate synthase (*TYMS*) 5′UTR and 3′UTR polymorphisms and methylenetetrahydrofolate reductase (*MTHFR*) C677T and A1298C polymorphisms were assessed in peripheral leukocytes. Univariate and multivariate analyses were applied to evaluate which variables could predict chemotherapy-induced toxicity, disease-free survival (DFS) and overall survival (OS). Multivariate analysis showed that: (a) low 5FU clearance was an independent predictive factor for severe toxicity (OR=7.32; *P*<0.0001); (b) high-5FU clearance predicted poorer DFS (HR=1.96; *P*=0.041) and OS (HR=3.37; *P*=0.011); (c) advanced age was associated with shorter DFS (HR=3.34; *P*=0.0008) and OS (HR=2.66; *P*=0.024); (d) the C/C genotype of the *MTHFR* C677T polymorphism was protective against grade 3–4 toxicity (*P*=0.040); (e) none of the *TYMS* polymorphisms could explain 5FU toxicity or clinical outcome.

Fluorouracil (5FU) is a fundamental component of all chemotherapic combinations for palliative (Board and Valle, 2007) and adjuvant treatments (Chung and Saltz, 2007) of colorectal cancer. Its main mechanism of action consists of inhibition of thymidylate synthase (TS) through an active metabolite, fluorodeoxyuridine monophosphate (FdUMP), which forms an inactive ternary complex with TS and 5–10 methylenetetrahydrofolate (MTHF) (Pinedo and Peters, 1988). Intracellular MTHF levels, which are essential for ternary complex stabilisation, are controlled by the enzyme methylenetetrahydrofolate reductase (MTHFR) (Scott and Weir, 1994). Consequently, the tissue activities of TS and MTHFR are presumed to be major determinants of 5FU clinical response. The activity of both enzymes is under genetic control and several gene polymorphisms are known to influence their tissue expression. A variable number of 28 bp tandem repeats (VNTR) is present in the 5′-untranslated region (5′-UTR) of the TS gene (*TYMS*). The allele containing the triple repeat (3R) is associated with 3–4-fold translational efficiency, compared with the double repeat allele (2R) (Kawakami *et al*, 2001). A second polymorphism has been identified in the second 28 bp repeat of 3R alleles, consisting of a G>C base change at the twelfth nucleotide, which makes the transcriptional activity of the 3R allele as low as that of the 2R allele (Mandola *et al*, 2003). Third, a 6 bp deletion may occur in the 3′-untranslated region (3′-UTR) of *TYMS*, associated with decreased mRNA stability and lower TS expression (Mandola *et al*, 2004). Two functionally important single nucleotide polymorphisms (SNP) have also been detected in the MTHFR gene (*MTHFR*), C677T and A1298C, resulting in decreased MTHFR activity (Ulrich *et al*, 2002).

Despite these theoretical and experimental premises, clinical studies which have evaluated the effect of *TYMS* (Gusella and Padrini, 2007) and *MTHFR* (Robien *et al*, 2005) polymorphisms on the colorectal cancer response/toxicity to 5FU have produced inconsistent results.

An additional determinant of 5FU activity is the metabolic rate of dihydropyrimidine dehydrogenase (DPD), the enzyme responsible for 5FU inactivation (van Kuilenburg, 2004). Variability in DPD activity is only marginally explained by genetic factors, as deficient variants of DPD gene are extremely rare (van Kuilenburg *et al*, 2001). Hence, DPD activity is best determined by direct *in vivo* measurement of 5FU plasma clearance (CL).

The aim of our work was to evaluate the clinical outcome and toxicity of colorectal cancer patients on adjuvant treatment with 5FU in relation with demographic characteristics, known *TYMS* and *MTHFR* polymorphisms, and individual 5FU CL.

## Materials and methods

### Patients and treatment

A total of 130 consecutive B2 and C Duke's stage colorectal cancer patients on adjuvant therapy were prospectively studied between 1999 and 2008. Chemotherapeutic treatment consisted of the 5FU+leucovorin combination according to the Mayo administration schedule (O'Connel *et al*, 1997) (2 min i.v. bolus administration of 425 mg m^–2^ 5FU+20 mg m^–2^ folinic acid daily for 5 days, for 6 consecutive cycles every 4 weeks). The starting dose could be reduced by 25% in the oldest patients. If toxicity of grade ⩾2 occurred, the next doses were reduced by 25–50% or stopped, according to toxicity level. The study was approved by the Ethics Committee of the Azienda–ULSS 18 Rovigo, and all patients gave their written informed consent.

### Clinical evaluation

Toxicity was recorded and graded according to World Health Organization (WHO) criteria and was dichotomously classified for statistical analysis in none-to-moderate (grade 0–2) and severe (grade 3–4).

The outcome variables were disease-free survival (DFS) and overall survival (OS). For DFS, an event was defined as the first occurrence of a tumour relapse that was not preceded by a second primary cancer. For OS, the outcome was death related to cancer and patients who died for other causes were treated as censored in survival analysis. The duration of follow-up was measured from the date of starting chemotherapy. Follow-up visits were scheduled every 3 months in the first year, every 6 months in the second and third years, and every year thereafter.

### Pharmacokinetic analysis

5FU pharmacokinetics was determined on day 2 of the first therapy cycle, using an HPLC method and a limited sampling strategy described earlier (Gusella *et al*, 2002). Plasma concentration decay was analysed with a mono-exponential function, so that intercept (A) and slope (k) were obtained and used to calculate the main pharmacokinetic parameters:

- area under the plasma concentration-time curve: AUC=A/k;
- plasma clearance: CL=Dose/AUC.

With the limited sampling methods used, bias and accuracy for AUC estimate are 3.4 and 5.1%, respectively (Gusella *et al*, 2002).

### Genetic analysis

Genomic DNA was extracted from peripheral blood leukocytes with a commercial kit (Promega Corporation, Madison, WI, USA) and stored at 4°C until genotyping. 0.2 *μ*g per sample of genomic DNA was used to carry out the PCR using specific primers in a 50 *μ*l final volume; genotyping was carried out according to earlier reported methods for TYMS 5′UTR VNTR and G>C substitution (Gusella *et al*, 2006a), TYMS 3′UTR 6 bp insert-deletion (Ulrich *et al*, 2000), MTHFR C677T and MTHFR A1298C polymorphisms (Miao *et al*, 2002).

Repeat genotyping of a 10% random sample yielded a 100% agreement.

### Classification of TS expression based on combined 5′UTR and 3′UTR genotypes

Variable number of tandem repeats and G>C SNP polymorphisms in the 5′UTR were classified according to Kawakami and Watanabe (2003) in low expression (2R/2R, 2R/3RC, 3RC/3RC) and high-expression TS genotypes (2R/3RG, 3RC/3RG, 3RG/3RG). 5′UTR and 3′UTR were also combined in two different ways, based on the expected TS expression level (see Table 1). The simplest combination (combination A) takes into account VNTR and ins/del 3′UTR polymorphisms and classifies TS activity as ‘high’ or ‘low’, according to Hitre *et al* (2005). The more complex combination (combination B) also includes G>C SNP in the 5′UTR and divides TS activity into three expression groups: ‘high’, ‘intermediate’ and ‘low’.

### Classification of MTHFR activity based on combined C677T and A1298C genotypes

Similarly, MTHFR activity was classified as ‘high’, ‘intermediate’ and ‘low’, based on the expected MTHFR metabolic rate of the combined C677T and A1298C polymorphisms (Ulrich *et al*, 2002) (see Table 2).

### Statistical analysis

Univariate logistic regression was used to identify the baseline prognostic factors related to toxicity. The factors significant at the 0.20 level were then introduced in a multivariate logistic regression model, and a backward-elimination approach was adopted. Interactions were tested at the 0.10 level of significance. The results are presented as odds ratio (OR) and 95% confidence interval.

The hazard ratios (HRs) for disease recurrence and mortality, were estimated for individual covariates with the univariate Cox proportional-hazards model. Variables significant at the 0.20 level in the univariate analysis were entered into multivariate Cox proportional-hazards models in a backward selection procedure. Interactions were tested at the 0.10 level of significance. Proportionality assumption was tested including time-dependent covariates in the final model at a significance level of 0.10. The results are presented as HR and 95% confidence interval. Covariates considered were age (two groups divided by the median value), sex, Dukes’ stage (B2 and C), *TYMS* VNTR polymorphism (three genotypes), *TYMS* G>C SNP polymorphism (low- and high-expression genotypes), *TYMS* ins/del polymorphism (three genotypes), *TYMS* 5′UTR and 3′UTR combined genotypes (combination A: two groups; combination B: three groups; see Table 1), *MTHFR* C677T polymorphism (three genotypes), *MTHFR* A1298C polymorphism (three genotypes), *MTHFR* 677 and 1298 combined genotypes (three groups; see Table 2), 5FU clearance per Kg (two groups divided by median value) and true cumulative 5FU dose administered, expressed as a percentage of the theoretical dose (continuous).

Survival curves were estimated using the Kaplan–Meier method and differences between individual curves were assessed by log-rank test. Data are presented as means±s.d., unless otherwise specified. The χ^2^-test and *t*-test for unpaired data were applied to compare frequencies and means, respectively. A one-way ANOVA (followed by the *post-hoc* linear-trend test, when needed) was used to compare groups. All tests were two-sided with a 5% significance level. Data were analysed with version 9.1.3 of SAS software for Windows (SAS Institute Inc., Cary, NC, USA).

Hardy–Weinberg equilibrium and linkage disequilibrium were evaluated by PyPop on line free software (University of California, Berkley, USA) (Lancaster *et al*, 2003).

## Results

### Clinical data

A total of 130 patients (46 women and 84 men) were the study population. All had a Karnofsky performance status score between 100 and 80. Mean age was 63.7±10.3, ranging from 34 to 84 years. The primary tumour site was right colon in 47 cases and left colon in 83 cases. Duke's tumour stage was B2 in 51 patients and C in 79 patients. Stage C was more frequently found in patients aged ⩾65 years than in those aged <65 (80.3 *vs* 42.2%; *P*<0.0001).

A total of 116 patients were treated for all six planned cycles; 10 patients stopped the treatment prematurely because of toxicity, mainly at the first or second cycle, and four patients stopped because of disease progression. The true cumulative dose was, on average, 74.1±20.2% (range: 14.4–101.6%) of the theoretical one, and dose reduction was significantly greater in patients aged ⩾65 (30.5±19.0%) than <65 (21.1±20.5%) (*P*<0.0075).

During chemotherapy, 45 (35%) patients suffered from grade 3–4 toxicity, mainly mucositis (28%), diarrhea (10%) and neutropenia (8%). During follow-up (median 3.8 years, range 0.4–8.5 years), 44 patients had documented recurrent disease and 31 died, three for non cancer-related causes. Overall survival rates were 87.6% at 3 years and 73.5% at 5 years. The 3- and 5-year DFS rates were 69.8 and 62.2%, respectively.

### Pharmacokinetic data

**Figure 1:**
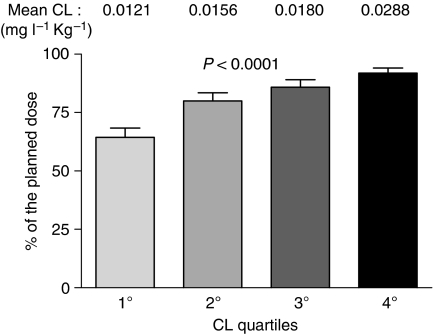
Mean cumulative 5FU dose administered, as percentage of initially planned dose, in each 5FU clearance quartile. Bars represent standard errors. Four patients who stopped therapy because of disease progression were excluded from analysis.

The mean 5FU starting dose was 724.5±88.2 mg (range: 350–920 mg) and the related pharmacokinetic parameters were broadly variable among patients (AUC range: 146–1236 mg l^–1^ × min; CL range: 0.0084–0.0705 l min^–1^ Kg^–1^). 5FU clearance measured at the first cycle was inversely correlated with the percent dose reduction during the following cycles: the lower the CL, the greater the reduction in the cumulative dose, with a linear trend across the CL quartiles (ANOVA, *P*<0.0001; linear trend, *P*<0.0001) (Figure 1).

### Genetic data

The frequency of *TYMS* and *MTHFR* genotypes found in our population was similar to that reported earlier in white populations, and allelic distributions were in Hardy–Weinberg equilibrium. Strong-linkage disequilibrium was found for 5′UTR VNTR and 3′UTR polymorphisms (*d*′=0.81; *P*<0.0001), 5′UTR G>C and 3′UTR polymorphisms (*d*′=0.52; *P*<0.0001) and *MTHFR* C677T and A1298C polymorphisms (*d*′=0.96, *P*<0.0001). *TYMS* and *MTHFR* genotype distributions were not significantly different between stages B2 and C, males and females, patients aged <65 and ⩾65 years.

### Toxicity analysis

Univariate analysis showed that low-5FU CL had a highly significant effect on severe toxicity (*P*<0.0001). Among the other variables, only age was marginally significant (*P*=0.059) (Table 3). Multivariate analysis confirmed low-5FU CL (*P*<0.0001) as an independent prognostic factor for grade 3–4 toxicity (OR=7.32, 95% CI: 3.06–17.51, *P*<0.0001); in addition, the ‘high-activity’ *MTHFR* 677 CC genotype proved to be associated with a reduced risk of side-effects (T/T *vs* C/C OR=1.17, 95% CI: 0.34–4.08; C/T *vs* C/C OR=3.10, 95% CI: 1.21–7.94, *P*=0.040). When specific toxicity type was considered, severe mucositis was predicted only by low 5FU CL (OR=7.92, 95% CI: 3.00–20.91, *P*<0.0001), whereas no variable predicted diarrhea and neutropenia.

### Survival analysis

Survival analysis was restricted to the 122 patients who completed at least three chemotherapy cycles, that is, those who received at least 50% of the planned cumulative dose, in order to exclude the patients whose clinical outcome could not be reasonably ascribed to 5FU treatment. Univariate analysis showed that advanced age and Dukes’ stage C had a significant negative prognostic effect on DFS, and that high-5FU CL was significantly related with a worse OS (Table 4). Conversely, sex, cumulative dose reduction and onset of severe toxicity had no predictive value. Also, no significant association was observed between DFS or OS and any *TYMS* or *MTHFR* polymorphisms, either analysed separately or in combination.

In the multivariate Cox-regression model, advanced age and high-5FU clearance proved to be unfavourable prognostic factors for both DFS and OS. An age ⩾65 was associated with an HR of 3.34 for relapsing (95% CI: 1.65–6.75; *P*=0.0008) and 2.66 for dying (95% CI: 1.32–8.64; *P*=0.024). Patients with CL ⩾0.017 l min^–1^ Kg^–1^ had a cancer-related death risk 3.37 times higher (95% CI: 1.32–8.64; *P*=0.011) and a disease-recurrence risk 1.96 times higher (95% CI: 1.03–3.73; *P*=0.041) than patients with CL <0.017 l min^–1^ Kg^–1^.

**Figure 2:**
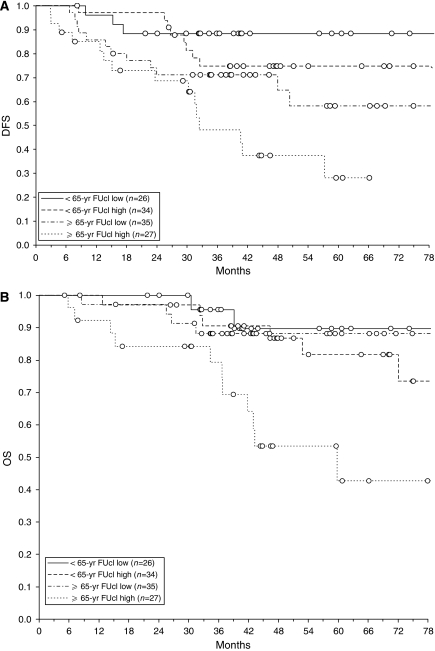
Disease-free (**A**) and overall survival (OS) plots (**B**) of patients stratified in four groups according to age (< or ⩾65 years) and 5FU clearance (< or ⩾0.017 mgl^−1^Kg^−1^). Open circles indicate censored data points. Log-rank test was significant for both disease-free survival (DFS) (*P*=0.0012) and overall survival (OS) (*P*=0.0036).

When patients were stratified according to CL and age in four groups (Figure 2), those with high CL and aged ⩾65 survived less (HR: 6.50; 95% CI: 1.44–29.36; *P*=0.015) and relapsed earlier (HR: 5.99; 95% CI: 1.95–18.51; *P*=0.0018) than those with low CL and aged <65, who had the best outcome.

A significant difference in DFS was also observed between old/high CL and young/high CL patients (log-rank test: *P*=0.025) and between old/low CL and young/low CL patients (*P*=0.047). In addition, OS of patients aged >65 and with high CL was significantly worse than that of any other group (*P* range: 0.012–0.009). The comparisons between the other groups did not reach the significance level.

Frequency of Duke's C stage was lower in the younger/low CL group than in the older/high CL group (42.3 *vs* 81.5%, *P*=0.0033), whereas severe toxicity was more frequent in the former group than in the latter (42.3 *vs* 18.5%, *P*=0.059). Nevertheless, as stated above, in the overall population neither C Duke's stage nor severe toxicity were markers of OS/DFS, as their predictive value in the multivariate analysis was probably included in age and CL, respectively.

## Discussion

The main clinical implication emerging from our study is that high-5FU CL (⩾0.017 l min^–1^ Kg^–1^), measured at the beginning of the adjuvant chemotherapy course in colorectal patients, is the strongest single predictor of reduced toxicity and poor clinical outcome in terms of both DFS and OS. Multivariate analysis also showed that age ⩾65 was associated with shorter DFS and OS but not with more severe toxicity, suggesting that preventive dose reduction in elderly patients is not advisable. As a whole, the association of high-5FU CL with advanced age had the worse prognostic meaning for both DFS and OS. Furthermore, patients homozygous C/C for C677T *MTHFR*, who are presumed to have-high enzyme activity, had a lower-toxicity risk.

With respect to 5FU tolerability, our findings are in good agreement with earlier literature data, as high AUC or low CL have invariably been associated with more severe 5FU toxicity after any administration schedule (Young *et al*, 1999; Di Paolo *et al*, 2002; Jansman *et al*, 2002; Ychou *et al*, 2003; Codacci-Pisanelli *et al*, 2005; Gusella *et al*, 2006b; Capitain *et al*, 2008). A recent, large, prospective trial (Schwab *et al*, 2008) on cancer patients treated with 5FU monotherapy confirmed a significant––although weak––link between mutant *MTHFR* C677T genotypes and FU toxicity but, at odds with our results, also found a significant correlation with the 2R/2R *TYMS* genotype. A correlation between 5FU toxicity and the VNTR *TYMS* polymorphism has also been reported by Lecomte *et al* (2004) and Pullarkat *et al* (2001), who found less toxicity in patients with the 3R/3R genotype. Indeed, our univariate analysis also suggests a non-significant trend toward a decreased toxicity risk in patients carrying the 3R/3R genotype in comparison with carriers of the 2R/2R *TYMS* genotype (OR=0.55). A significant correlation with C677T MTHFR genotype, but not with TYMS genotypes, was also found by Sharma *et al* (2008) in colorectal patients treated with capecitabine, a 5FU prodrug.

With respect to factors correlated with therapeutic outcomes, age and 5FU CL turned out to be the only significant predictors of DFS and OS in our study. The association between age and clinical outcome may be partly explained by the greater prevalence of stage C in our older patients. However, other studies found that advanced age is an independent risk factor (Gill *et al*, 2004; Schippinger *et al*, 2007). Similarly, an association between low 5FU CL or high AUC and improved 5FU response has already been reported in cancer patients treated with either continuous 5FU infusion (Milano *et al*, 1994; Gamelin *et al*, 1996) or Machover's bolus administration schedule (Di Paolo *et al*, 2008).

DPD governs 5FU catabolism in both normal and tumour cells, and a significant correlation has been shown between DPD expression in colorectal tumours and the surrounding intestinal mucosa (Tanaka-Nozaki *et al*, 2001; Amatori *et al*, 2006). Thus, on the one hand, a low-5FU CL may improve 5FU delivery to the tumour and, on the other, decrease 5FU inactivation inside it. In line with this view, several studies in colorectal cancer patients treated with 5FU-based adjuvant therapy have shown that low-DPD protein or mRNA expression in the tumour is associated with longer DFS and OS (Popat *et al*, 2004; Ciaparrone *et al*, 2006; Jensen *et al*, 2006; Lassmann *et al*, 2006; Popat *et al*, 2006; Yamada *et al*, 2008), whereas no consistent correlation has been shown with TS tumour expression (Kamoshida *et al*, 2004; Aguiar *et al*, 2005; Ciaparrone *et al*, 2006; Jensen *et al*, 2006; Lassmann *et al*, 2006; Popat *et al*, 2006; Soong *et al*, 2008; Yamada *et al*, 2008). Indeed, in our study, none of the three TS polymorphisms, considered alone or in combination (VNTR, G>C substitution, 6 bp insertion-deletion), could predict the clinical outcome. This negative results may be because of an insufficient power of our study to detect genetic associations or to some inaccuracy in grouping the genotypes. However, our population size was comparable with that of other similar studies and genotype classifications reflected the best available information on gene biological activity. In this respect, literature data are inconclusive in that the same TS polymorphisms have been correlated with both favourable and unfavourable effects, and no correlation has even been reported (Dotor *et al*, 2006; Gusella and Padrini, 2007; Gosens *et al*, 2008; Fernandez-Contreras *et al*, 2009). Besides the methodological differences between clinical trials, a number of biological factors may have obscured the link between *TYMS* polymorphisms and 5FU activity. First, it is known that 5FU can not only inhibit TS but also affect mRNA function. Sobrero *et al* (1997) have suggested, and others (Kubota *et al*, 2002; Matsusaka *et al*, 2003; Hoshino *et al*, 2005) have experimentally confirmed, that 5FU incorporation into colorectal tumour mRNA is significantly greater after bolus than after continuous infusion. If this is true, RNA-mediated effects may prevail over TS inhibition, and TS expression may play only an ancillary role in the antitumour activity of 5FU bolus administration. Second, translation of TS mRNA is inhibited by TS protein in an autoregulatory manner (Chu *et al*, 1991), so that the influence of gene polymorphisms may be blunted. Third, it has been reported that 5FU treatment can induce a variable, compensatory increase in TS mRNA levels in colorectal tumours (Ukida *et al*, 2001; Tanaka-Nozaki *et al*, 2003) and that patients with no or minor mRNA increase show a better response to therapy (Ukida *et al*, 2001). Fourth, the 5′-UTR polymorphism is in linkage disequilibrium with the 3′-UTR polymorphism, in that the +6/+6 bp and −6/−6 bp genotypes are associated with 2R/2R and 3R/3R genotypes, respectively (Mandola *et al*, 2004). It is therefore likely that the coexistence in the same gene of high- and low-expression genotypes may attenuate functional consequences. Fifth, TS tumour levels may be simultaneously markers of tumour aggressiveness and tumour sensitivity to 5FU. In this regard, a recent, large study (Soong *et al*, 2008) reported that low-TS expression was correlated with worse prognosis in colorectal patients treated with surgery alone whereas no significant effect on survival was noted in the 5FU-treated group. As it is generally acknowledged that low TS-expressing tumours are more sensitive to 5FU (Gusella and Padrini, 2007), the lack of prognostic significance of low TS levels in 5FU-treated patients may be explained by a concomitant harmful effect on tumour growth.

The prognostic role of the *MTHFR* polymorphism in the response to 5FU did not emerge from our study. No studies with adjuvant therapy are available for a comparison, although contradictory results come from studies in other clinical settings (Cohen *et al*, 2003; Etienne *et al*, 2004; Jakobsen *et al*, 2005; Terrazzino *et al*, 2006; Ruzzo *et al*, 2007; Zhang *et al*, 2007). The reasons for such discrepancies are unclear.

In conclusion, our study makes a contribution to the controversial issue of which colorectal patients are more likely to gain benefit from 5FU adjuvant chemotherapy. One strength of our investigation is the simultaneous evaluation of an index of drug exposure (5FU CL) and of several *TYMS* and *MTHFR* polymorphisms possibly related to tumour sensitivity, in a group of patients homogeneously treated with a repeated bolus regimen. The finding that patients’ demographic and pharmacokinetic characteristics are more predictive of clinical outcome than some putative genetic markers suggests that the traditional, phenotypic approach to clinical cancer research should not be abandoned and that the genetic approach requires further work to prove its clinical usefulness. Feasibility and utility of a pharmacokinetic test to assess 5FU CL in colorectal patients starting adjuvant therapy need to be evaluated in a prospective trial.

## Figures and Tables

**Table 1:** *TYMS* combined genotypes classified according to expected expression of thymidylate synthase

	**Expression level**	***TYMS* 5′UTR**	***TYMS* 3′UTR**	***n***
G>C SNP (Kawakami and Watanabe, 2003)	High	2R/3RG,3RC/3RG, 3RG/3RG	—	58
	Low	2R/2R, 2R/3RC, 3RC/3RC	—	72
				
Combination A (Hitre *et al*, 2005)	High	3R3R	Any genotype	61
		2R3R	ins/ins	
	Low	2R3R	ins/del–del/del	69
		2R2R	any genotype	
				
	High	2R/3RG,3RC/3RG, 3RG/3RG	ins/ins	8
Combination B	Intermediate	2R/3RG,3RC/3RG, 3RG/3RG	ins/del–del/del	88
		2R/2R, 2R/3RC, 3RC/3RC	ins/ins	
	Low	2R/2R, 2R/3RC, 3RC/3RC	ins/del–del/del	34

Abbreviations: SNP=single nucleotide polymorphism; UTR=untranslated region.

**Table 2:** *MTHFR* combined genotypes classified according to expected activity of metylenetetrahydrofolate reductase

**Activity level**	***MTHFR* C677T**	***MTHFR* A1298C**	***N***
High	C/C	A/A	10
Intermediate	C/C	C/C, A/C	78
	T/T, C/T	A/A	
Low	T/T, C/T	C/C, A/C	42

Abbreviation: MTHFR=methylenetetrahydrofolate reductase.

**Table 3:** Univariate logistic regression of toxicity in 130 patients adjuvantly treated with 5FU chemotherapy

		**TOXICITY**			
	***n***	**Events**	***P***	**OR**	**95% CI**
*Sex*					
Female	46	17	0.68	1	—
Male	84	28		0.85	0.40–1.81
					
*Age (years)*					
<65	64	17	0.06	1	—
⩾65	66	28		2.04	0.97–4.26
					
*TYMS VNTR*					
2R2R	25	10	0.38	1	—
2R3R	60	23		0.93	0.36–2.42
3R3R	45	12		0.55	0.19–1.54
					
*TYMS G>C*					
Low activity	72	25	0.98	1	—
High activity	58	20		0.99	0.48–2.047
					
*TYMS 3′UTR*					
ins/ins	46	20	0.10	1	—
ins/del	68	23		0.66	0.31–1.43
del/del	16	2		0.19	0.04–0.91
					
*TYMS comb A*					
Low activity	69	25	0.68	1	—
High activity	61	20		0.86	0.41–1.77
					
*TYMS comb B*					
Low	34	11	0.09	1	—
Intermediate	88	28		0.98	0.42–2.28
High	8	6		6.27	1.09–36.22
					
*MTHFR C677T*					
C/C	44	11	0.08	1	—
C/T	63	28		2.40	1.03÷5.58
T/T	23	6		1.06	0.33–3.36
					
*MTHFR A1298C*					
A/A	54	22	0.12	1	—
A/C	59	21		0.80	0.38–1.72
C/C	17	2		0.19	0.04–0.93
					
*MTHFR combined*					
Low	42	16	0.75	1	—
Intermediate	78	25		0.77	0.35–1.68
High	10	4		1.08	0.26–4.44
					
*5FU CL (lmin^−1^Kg^−1^)*					
⩾0.017	65	10	<0.0001	1	—
<0.017	65	35		6.42	2.79–14.74
					

Abbreviations: CI=confidence interval; CL=plasma clearance; MTHFR=methylenetetrahydrofolate reductase; OR=odds ratio; UTR=untranslated region; VNTR=variable number tandem repeats.

**Table 4:** Univariate Cox regression of disease free and overall survival in the 122 patients adjuvantly treated with 5FU chemotherapy for at least three cycles

		**DFS**				**OS**			
	***n***	**Events**	***P***	**HR**	**95% CI**	**Events**	***P***	**HR**	**95% CI**
*Sex*									
Female	41	13	0.86	1	—	7	0.60	1	—
Male	81	27		1.06	0.55–2.06	17		1.26	0.52–3.05
									
*Age (years)*									
<65	60	13	0.003	1	—	9	0.08	1	—
⩾65	62	27		2.90	1.46–5.79	15		2.12	0.91–4.91
									
*Dukes’ stage*									
B2	46	10	0.04	1	—	6	0.16	1	—
C	76	30		2.10	1.02–4.30	18		1.94	0.77–4.89
									
*Toxicity grade*									
0–2	81	25	0.43	1	—	17	0.68	1	—
3–4	41	15		1.30	0.68–2.47	7		0.83	0.34–2.01
									
*TYMS VNTR*									
2R2R	23	11	0.24	1	—	7	0.34	1	—
2R3R	57	18		0.63	0.30–1.35	12		0.72	0.28–1.83
3R3R	42	11		0.49	0.21–1.14	5		0.42	0.13–1.34
									
*TYMS G>C*									
Low activity	68	23	0.79	1	—	15	0.58	1	—
High activity	54	17		0.92	0.49–1.72	9		0.79	0.35–1.81
									
*TYMS 3′UTR*									
ins/ins	44	13	0.65	1	—	8	0.67	1	—
ins/del	63	24		1.29	0.66–2.53	15		1.30	0.55–3.06
del/del	15	3		0.84	0.24–2.98	1		0.59	0.07–4.80
									
*TYMS comb. A*									
Low	64	25	0.15	1	—	16	0.22	1	—
High	58	15		0.62	0.33–1.18	8		0.58	0.24–1.38
									
*TYMS comb. B*									
Low activity	32	12	0.69	1	—	8	0.58	1	—
Intermediate	82	26		0.74	0.37–1.48	15		0.64	0.27–1.50
High activity	8	2		0.71	0.16–3.17	1		0.64	0.08–4.95
									
*MTHFR C677T*									
C/C	41	13	0.54	1	—	8	0.86	1	—
C/T	59	18		0.85	0.42–1.74	11		0.83	0.33–2.06
T/T	22	9		1.34	0.57–3.14	5		1.07	0.35–3.27
									
*MTHFR A1298C*									
A/A	49	17	0.73	1	—	9	0.86	1	—
A/C	57	19		0.82	0.42–1.60	13		1.01	0.43–2.38
C/C	16	4		0.68	0.23–2.02	2		0.67	0.14–3.10
									
*MTHFR comb.*									
Low	41	12	0.30	1	—	9	0.95	1	—
Intermediate	72	26		1.62	0.81–3.24	13		1.10	0.46–2.59
High	9	2		0.83	0.18–3.72	2		1.28	0.27–5.98
									
*CL (l min* ^*–1*^ * Kg* ^*–1*^ *)*									
<0.017	61	16	0.17	1	—	6	0.032	1	—
⩾0.017	61	24		1.56	0.83–2.95	18		2.76	1.09–6.99
									
% Dose reduction	Continuous		0.16	1.02	0.99–1.04		0.27	1.02	0.99–1.04
									

Abbreviations: CI=confidence interval; CL=plasma clearance; DFS=disease-free survival; HR=hazard ratio; MTHFR=methylenetetrahydrofolate reductase; OS=overall survival; UTR=untranslated region; VNTR=variable number tandem repeats.
